# A saliva-based rapid test to quantify the infectious subclinical malaria parasite reservoir

**DOI:** 10.1126/scitranslmed.aan4479

**Published:** 2019-01-02

**Authors:** Dingyin Tao, Brent McGill, Timothy Hamerly, Tamaki Kobayashi, Prachi Khare, Amanda Dziedzic, Tomasz Leski, Andrew Holtz, Bruce Shull, Anne E. Jedlicka, Andrew Walzer, Paul D. Slowey, Christopher C. Slowey, Sandrine E. Nsango, David A. Stenger, Mike Chaponda, Modest Mulenga, Kathryn H. Jacobsen, David J. Sullivan, Sadie J. Ryan, Rashid Ansumana, William J. Moss, Isabelle Morlais, Rhoel R. Dinglasan

**Affiliations:** 1 W. Harry Feinstone Department of Molecular Microbiology and Immunology, Johns Hopkins Bloomberg School of Public Health, Baltimore, MD 21205, USA; 2 Johns Hopkins Malaria Research Institute, Baltimore, MD 21205, USA; 3 Emerging Pathogens Institute and Department of Infectious Diseases & Immunology, College of Veterinary Medicine, University of Florida, Gainesville, FL 32611, USA; 4 Department of Epidemiology, Johns Hopkins Bloomberg School of Public Health, Baltimore, MD 21205, USA; 5 United States Naval Research Laboratory (NRL), Center for Biomolecular Science and Engineering, Washington, DC 20375, USA; 6 College of Science, George Mason University, Fairfax, VA 22030, USA; 7 Thermo Fisher Scientific, Fremont, CA 94538, USA; 8 Oasis Diagnostics, Vancouver, WA 98686, USA; 9 Laboratoire de Recherche sur le Paludisme, Institut de Recherche pour le Développement–Organisation de Coordination et de Coopération pour la Lutte Contre les Grandes Endémies en Afrique Centrale (IRD-OCEAC), Yaoundé, Cameroon; 10 Faculty of Medicine and Pharmaceutical Sciences, University of Douala, PO Box 2701, Douala, Cameroon; 11 Tropical Disease Research Centre, Ndola, Zambia; 12 College of Health and Human Services, George Mason University, Fairfax, VA 22030, USA; 13 Emerging Pathogens Institute and Department of Geography, College of Liberal Arts and Sciences, University of Florida, Gainesville, FL 32611, USA; 14 Mercy Hospital Research Laboratory, Kulanda Town, Bo, Sierra Leone

## Abstract

A large proportion of ongoing malaria parasite transmission is attributed to low-density subclinical infections not readily detected by available rapid diagnostic tests (RDTs) or microscopy. *Plasmodium falciparum* gametocyte carriage is subclinical, but gametocytemic individuals comprise the parasite reservoir that leads to infection of mosquitoes and local transmission. Effective detection and quantification of these carriers can help advance malaria elimination strategies. However, no point-of-need (PON) RDTs for gametocyte detection exist, much less one that can perform noninvasive sampling of saliva outside a clinical setting. Here, we report on the discovery of 35 parasite markers from which we selected a single candidate for use in a PON RDT. We performed a cross-sectional, multi-omics study of saliva from 364 children with subclinical infection in Cameroon and Zambia and produced a prototype saliva-based PON lateral flow immunoassay test for *P. falciparum* gametocyte carriers. The test is capable of identifying submicroscopic carriage in both clinical and nonclinical settings and is compatible with archived saliva samples.

## INTRODUCTION

Malaria kills ~500,000 children each year, mostly children under the age of 5 in sub-Saharan Africa (*1*). Although malaria control efforts are having a substantial impact on disease-related deaths, parasite transmission remains a major issue. Low-density subclinical infection with *Plasmodium falciparum* among defined population subsets has been shown to be an important but cryptic facet of malaria transmission. Typically, these individuals are not readily detected by currently available point-of-need (PON) rapid diagnostic tests (RDTs) or microscopy (*2**–**5*), and molecular methods not readily available to hospitals, clinics, or other PON sites such as households, schools, or apothecaries are required for detection and quantification of parasite carriage. The sexual stage gametocytes do not cause disease, but gametocytemic individuals with asexual stages can eventually progress to clinical malaria, especially younger children. This subclinical gametocytemic population represents the parasite reservoir that drives local malaria transmission through mosquitoes. However, in both microscopy-detectable and submicroscopic subclinical infections, gametocytes are coincident with low-density asexual stages (*5*) and can contribute to up to 80% of the infectious reservoir (*5*). It is now well recognized that effective detection of these carriers, as a whole, is crucial to advancing malaria control and elimination strategies (*6**–**10*), especially in the context of low-transmission and pre-elimination settings. However, no point-ofcare or PON diagnostic for gametocyte detection exists (*11*).

The most commonly used sensitive and heat-stable blood-based RDT in sub-Saharan Africa is dependent on antibody detection of *P. falciparum* histidine-rich protein 2 (HRP2). Recently, in response to the growing concern that parasites lacking the *pfhrp2/pfhrp3* genes are resulting in false-negative HRP2-RDT results and children being left untreated, the World Health Organization (WHO) has encouraged the scientific community to identify new target biomarkers of the parasite that allow for the sensitive detection of carriage (*12*). To address this critical scientific gap and obstacle to malaria elimination and eradication, we developed a noninvasive diagnostic amenable for epidemiological surveillance programs and mass screening campaigns, both of which go beyond the limitation of a clinical setting. The use of blood for screening campaigns substantially increases the inherent risk to both the screener and the individual (patient), and complicates the logistics, training requirements, and efficiency of the surveillance effort. Recently, it has been shown that parasite or pathogen biomarkers can enter into other biofluids, specifically urine and saliva (*13**–**15*). Stage V, mature gametocytes circulate in the bloodstream but can also be sequestered in capillary beds (*16**–**18*). The oral cavity is highly vascularized with numerous capillary loops, and there is a dynamic transfer of serum transudate proteins from the vasculature as part of the gingival crevicular fluid (GCF) into the oral fluid (a mixture of saliva and GCF) in both healthy and diseased individuals (*19*). Here, we refer to oral fluid collection as saliva collection. We tested the hypothesis that if mature gametocytes potentially aggregate in gum capillary beds, then we can expect to sample gametocyte-derived proteins in the saliva of children infected with subclinical parasite densities in malaria-endemic countries such as Cameroon and Zambia.

Here, we report the identification of 35 *P. falciparum* proteins from carriers with subclinical parasitic infection, representing various stages of the parasite life cycle including stage V gametocytes, and a marker that is specific to mature female gametocytes. We performed a competitive profiling study using a liquid chromatography–multiple reaction monitoring (LC-MRM) mass spectrometry (MS) approach to estimate the prevalence of the candidate marker in the saliva of children with subclinical infection in Cameroon and Zambia and compared to microscopy and polymerase chain reaction (PCR) analyses of matched blood samples. Last, we developed a prototype antibody-based lateral flow immunoassay (LFIA) rapid test that further validated the presence of the marker in saliva from children with subclinical infection, revealing an underappreciated amount of subclinical carriage in these two malaria-endemic countries.

## RESULTS

### Protein marker discovery in the saliva of children with subclinical parasitic infection

We collected ethanol-stabilized saliva (5 ml) from 12 children (ages 5 to 12) in Mfou, Cameroon, who had subclinical parasitemia. Using a simple saliva collection strategy and LC-MS/MS approach, we generated a *P. falciparum* candidate protein marker list (Table 1 and tables S1 and S2). The proteins can be divided into several classes, including cytosolic “housekeeping” proteins that are conserved between asexual and gametocyte stages of *P. falciparum* (*20*), and those that are specific to asexual stages alone (*21**,* *22*). We noted that 22 of the proteins have been previously identified as highly enriched in stage V mature gametocytes (*19**,* *23*).

**Table 1:** **The most abundant *P. falciparum* proteins identified in children aged 5 to 16 years with subclinical parasitemia in Cameroon**. A total of 35 proteins were identified by LC-MS/MS with a Mascot ions score of ≥25 (table S1). Of the 35, the 19 proteins below were considered the most abundant. Score indicates the Mascot ions scores, as described previously *(**23**).* The *P. falciparum* female gametocyte-specific biomarker, indicated in bold, was found to have the highest Mascot ions score in our pooled sample. M_r_, molecular mass in kilodaltons (kDa).

Gene ID	Description	*M_r_* (kDa)	Annotated Gene Ontology	MS*	Mascot ions score
PF3D7_0401900	Acyl-CoA synthetase	110.8	Long-chain fatty acid-CoA ligase activity	A	28
PF3D7_0422300	Alpha tubulin 2	49.7	GTP binding, GTPase activity, structural molecule	A	31
PF3D7_0507800	Conserved *Plasmodium* protein	177.4	None	A, G	40
PF3D7_0509400	RNA polymerase I	340.7	DNA binding, DNA-directed RNA polymerase activity	G	47
PF3D7_0610400	Histone H3	15.5	DNA binding	A	534
PF3D7_0705500	Inositol-phosphate phosphatase, putative	330.7	None	G	31
PF3D7_0906100	Developmental protein, putative	21.9	None	A, G	37
PF3D7_1102400	Flavoprotein, putative	78.5	None	A	27
PF3D7_1134700	DNA-directed RNA polymerase 1, subunit 2, putative	175.5	DNA binding, DNA-directed RNA polymerase activity, ribonucleoside binding	G	37
PF3D7_1215100	Conserved *Plasmodium* protein	113.0	ATP binding, actin binding, calmodulin binding, motor activity	IG	27
PF3D7_1216900	DNA binding chaperone, putative	111.1	DNA binding, heat shock protein binding	A	36
**PF3D7_1218800**	**Conserved *Plasmodium* sexual stage protein, putative**	**39.6**	**None**	**G**	**949**
PF3D7_1235700	ATP synthase subunit beta, mitochondrial	58.4	Hydrogen ion transporting ATP synthase activity, rotational mechanism	A	116
PF3D7_1313500	Conserved *Plasmodium* membrane protein	209.1	Hydrolase activity, triglyceride lipase activity	G	42
PF3D7_1319200	Conserved *Plasmodium* protein	103.9	Flavin adenine dinucleotide binding	G	33
PF3D7_1325900	Conserved *Plasmodium* protein	326.2	ATP binding, actin binding, calmodulin binding, motor activity	G	34
PF3D7_1337500	Conserved *Plasmodium* protein	393.3	Calcium ion binding, receptor activity	IG	33
PF3D7_1353000	Tryptophan-rich antigen, pseudogene	96.3	None	A	49
PF3D7_1411400	Plastid replication- repair enzyme	235.8	3'-5' Exonuclease activity, ATP binding, DNA binding, DNA helicase/polymerase activity	G	31

* MS evidence for mature gametocytes (G), immature gametocytes (IG), and asexual stages (A), based on the following published references (*20*, *23*).

Of the 35 proteins that we detected in pooled saliva from the children, PF3D7_1218800 was the most abundant in individual saliva samples from children who were found to be gametocyte positive by blood film microscopy (Mascot ions scores; Table 1 and table S2). We had previously characterized PF3D7_1218800 as a female-specific stage V gametocyte marker (*23*) and corrected its current annotation to *Plasmodium* sexual stage protein 17 (PSSP17). We selected PSSP17 as a candidate gametocyte marker and developed an LC-MRM MS workflow to detect PSSP17 in saliva (2 ml) from children with subclinical infection to estimate the prevalence of marker carriage in children. The 2-ml volume was selected because there was no a priori information on the potential variation in the abundance of any parasite protein in the saliva of individuals with subclinical infection.

**Fig. 1:**
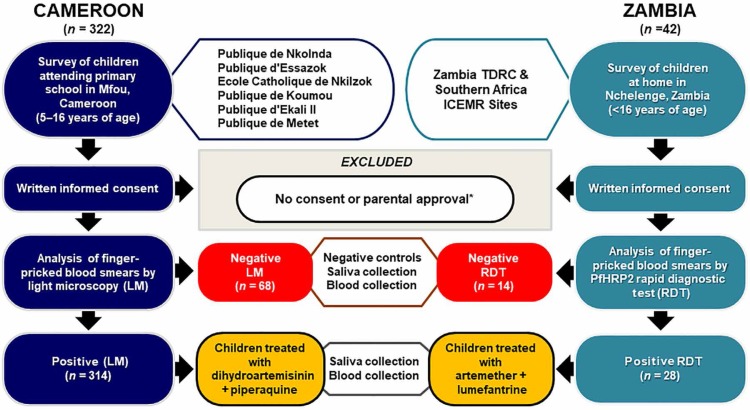
**Study design of the cross-sectional studies of subclinical malaria parasite carriage among children in Cameroon and Zambia**. Primary schools in the catchment area in Mfou, Cameroon, where screening and sampling performed are listed. School children between the ages of 5 to 16 were enrolled in the study. For Zambia, children <16 years of age who were present in households were sampled. The sampling strategy in Zambia dovetailed on an ongoing Southern Africa International Center of Excellence for Malaria Research (ICEMR) program project in Nchelenge, Zambia, which was conducted in partnership with the Tropical Diseases Research Centre (TDRC) of Zambia. Informed consent for all enrolled children was provided by a parent/guardian. If a child was found to be positive for malaria parasites by blood smear, they were referred for treatment according to the National Malaria Control Program guidelines. *Children who were not approved by their parent or guardian to participate were not included during the screen, and these children were not included in the total number of samples that were used for downstream molecular analyses.

We returned to Mfou, Cameroon, and sampled saliva from 307 children with subclinical infection (5 to 16 years of age, Fig. 1) attending six primary schools. In parallel, we sampled saliva from 42 children with subclinical malaria infection (under 16 years of age; table S3A) at home in Nchelenge District, Zambia (table S4). Three adults with subclinical infection (teachers from three different schools: Publique d’Ekali II, Publique de Metet, and Ecole Catholique de Nkilzok) were included in the blinded samples before analyses by LC-MRM MS, giving rise to a total of 310 samples from Cameroon during the second cross-sectional study. The total number of saliva samples collected and analyzed for both the initial and follow-up studies was 364. For our comparative profiling study, we acquired matched blood samples for blood film, RDT, and quantitative PCR (qPCR) analysis in Cameroon and matched blood samples for RDT and dried blood spot (DBS) cataloging and downstream PCR analysis in Zambia. These matched samples were important for comparing the sensitivity of current RDTs and orthogonal molecular detection techniques with MRM MS.

### Cross-sectional MS-based profiling of PSSP17 in saliva

**Fig. 2:**
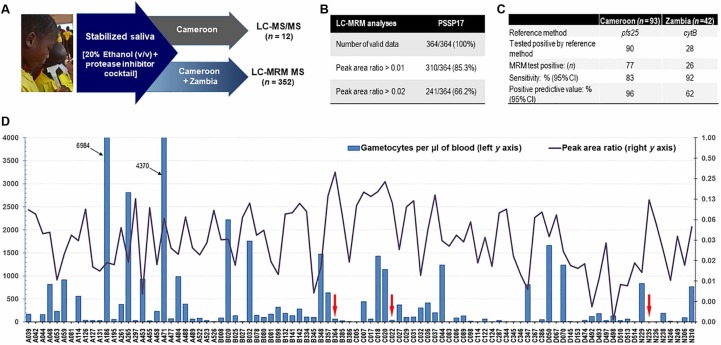
**Saliva and matched blood sample collection, stabilization, and analyses**. (**A**) Photograph and diagram of saliva collection (drool method) and stabilization for transport for two different collection periods from schools (Cameroon) and households (Zambia). The total number of saliva samples is 364. Saliva samples were stabilized for transport and analysis by LC-MS/MS for marker discovery. The prevalence of the marker PSSP17 in the saliva of children was then measured by LC-MRM MS. (**B**) Table summary of the LC-MRM results and estimated prevalence of marker carriage across all samples collected from both Cameroon and Zambia. (**C**) Table summary data of samples comparing the LC-MRM results with the reference molecular method of qPCR and PCR for *pfs25*, 18*S* rRNA gene in Cameroon, and *cytB* in Zambia, respectively. (**D**) Comparison of estimated female gametocytes/µl of blood (as determined by *pfs25* mRNA qPCR, left y axis, blue bars) and PSSP17 (PF3D7_1218800) protein expression (as determined by PAR, right *y* axis, gray bars) for a subset of 93 samples with paired qPCR (*pfs25*) and PAR data available. Sample case codes are indicated on the *x* axis. Red arrows refer to examples of discordant results, as described in the main text.

The blinded LC-MRM analyses of 364 stabilized samples identified 364 valid spectral data (Fig. 2A). Of the 364, we observed a high prevalence (85.3%, 310/364) of PSSP17 in the 5 to 16 years old age group in both Cameroon and Zambia, using a peak area ratio (PAR) of >0.01, with an average original peptide peak signal/noise (S/N) ratio of >10 (Fig. 2B). Using a more rigid cutoff of PAR > 0.02, with the average original peptide peak S/N > 15, the prevalence of carriage was 66.2% (241/364). In Cameroon, previous analyses of gametocyte carriage assessed by microscopy suggested that gametocytemic carriage in the same age group of children is about 6%, at a detection limit of ~8 gametocytes/µl of blood (*24**,* *25*). A summary of the unblinded comparative profiling study conducted in Cameroon and Zambia suggests, as expected, that the LC-MRM analyses showed strong agreement with microscopy and molecular assays for the detection of either female gametocytes (*pfs25* transcripts) or the presence of *Plasmodium* blood-stage parasites (asexual or gametocyte) via *Plasmodium* 18*S* ribosomal RNA (rRNA) from whole blood or cytochrome B (*cytB*) gene from DBS (Fig. 2C). For samples from Cameroon, we verified indirectly the presence of gametocytes by quantifying the gametocyte-specific transcript *pfs25* in a subset of 100 matched blood samples. Within this set were samples that were negative and positive by microscopy (submicroscopic carriage of gametocytes). The qPCR analyses confirmed gametocyte carriage in those individuals whose saliva samples were positive for PSSP17 by LC-MRM (table S5), with the exception of two samples: A042 and A081. It is unclear whether the RNA extraction procedure contributed to detection failure or whether gametocyte density was below the level of detection in our system. For those cases from Zambia where RDT detection failed (14/42), both PCR and LC-MRM analyses indicated parasite carriage. On the basis of our *cytB* nested PCR results, about 67% (28/42) were positive for parasite carriage (not gametocyte alone), whereas MRM confirmed the presence of gametocytes in 62% (26/42) of these samples, of which 67% (28/42) were also PCR positive.

We further analyzed a subset of samples (*n* = 93) with matched qPCR and LC-MRM data (Fig. 2D). We noted discrepancies in the estimated protein abundance (represented by PAR) and qPCR for samples B364, C022, and N235 (Fig. 2D, red arrows). For these three samples, despite the relatively lower gametocyte abundance as estimated by qPCR, PSSP17 appeared to be abundant in saliva. This is especially true for sample N235, where the *pfs25* qPCR estimated ~0.12 gametocytes/µl of blood, yet the protein marker was easily detected and measured in saliva. Conversely, five samples showed a marked inverse relationship between PAR and gametocytes/µl of blood estimates: A048, A265, A471, B020, and D070. The comparison in PSSP17/parasite detection approaches for the remaining available samples (*n* = 195) is shown in table S3B.

To test the relationship between PAR and gametocyte abundance [gametocytes/µl of blood (GAM)] for all data included in Fig. 2D, we first converted the PAR into binary positive and negative findings, using the LC-MRM cutoff value of 0.01 to define negative results. We then recoded GAM similarly. As expected, given the biology of gametocyte development, a χ^2^ test revealed no dependence of PAR on GAM (χ^2^ = 5.95 × 10^−31^, *P* = 1.00). For the raw continuous data, Shapiro-Wilk tests of normality were rejected for both PAR and GAM; thus, we used a nonparametric paired Wilcoxon signed-rank test with continuity correction (*V* = 36, *P* < 1.0 × 10^−16^). On the basis of these results, we reject the hypothesis that similarly ranked scores of the measures are the same. These data are visualized in fig. S1. These two tests tell us that there is no detectable connection or dependency between the PAR and GAM, as measured in this study.

### Development of a PSSP17 LFIA

**Fig. 3:**
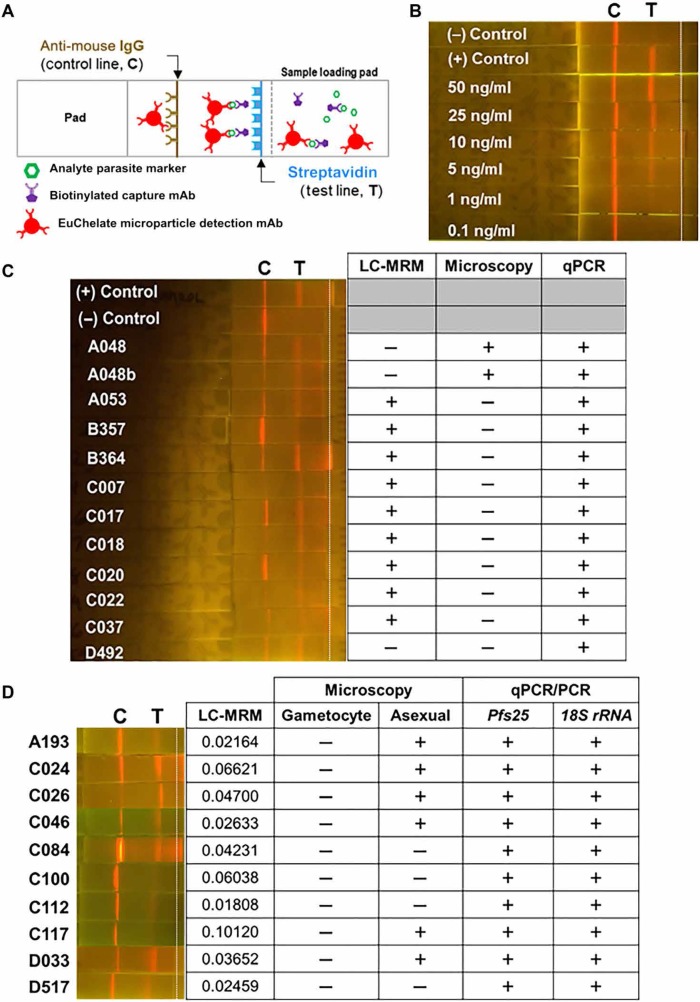
**Prototype LFIA RDT for PSSP17 (PF3D7_1218800)**. (**A**) Schematic of the gRAD lateral flow platform and the capture and detection of PSSP17 by EuChelate microparticle-conjugated mAbs. (B) Images of LFIA gRAD strips to estimate the LOD of recombinant PSSP17 in spiked-in assays using naïve human saliva as matrix. (**C**) Initial analyses of frozen, stabilized samples from Cameroon (*n* = 12). Samples were randomly selected and, upon unblinding, were found to be either positive or negative by MS (LC-MRM), microscopy, or qPCR. A048 and A048b are two independent saliva samples collected from the same child to show consistency in detection by the LFIA. Positive (+) control: gametocyte lysate spiked into naïve saliva; negative (−) control: irrelevant asexual parasite lysate spiked into naïve saliva. Images on the left side of the figure are gRAD platform strips that were captured by mobile phone camera. (**D**) A second subset of independent Cameroon samples (*n* = 10), positive by LC-MRM, was selected and compared across the same categories as in (C). Microscopy was subdivided into gametocyte versus asexual trophozoite positivity/negativity, and qPCR/PCR analyses were subdivided into gametocyte-specific transcript (*pfs25*) or a transcript present in both trophozoites and gametocytes (18*S* rRNA). For (B) to (D), 10 µl of saliva containing either recombinant PSSP17 or endogenous marker was used with each test. C, control line; T, test line. Vertical dotted line: Demarcation of the end of the sample loading pad of the gRAD strip.

To further confirm that MRM detection did not represent false positives or false negatives (due to the loss of its diagnostic potential during sample processing or because gametocyte abundance was below the limit of LC-MRM detection), we developed an orthogonal assay. Our prototype saliva-based LFIA test for PSSP17 uses LFIA strips, which contain a generic test line (streptavidin) and a control line [anti-mouse immunoglobulin G (IgG)] (Fig. 3A). We produced two high-affinity, murine monoclonal IgG antibodies to recombinant PSSP17, 10E2.B7 and 27C9.B5, for capture and detection, respectively. To complement the features of the LFIA strips and to allow for sensitive detection of the anticipated low amounts of circulating PSSP17, the monoclonal antibody (mAb) 10E2.B7 was biotinylated, and 27C9.B5 was conjugated to a Europium chelate (EuChelate) microparticle (*26*). EuChelate is readily excitable by a handheld ultraviolet light-emitting diode flashlight and exhibits visible fluorescence in normal lighting without requiring instrumentation. We selected the initial limit of detection (LOD) for our generic rapid assay device (gRAD) platform as 50 pg/ml recombinant PSSP17 using laboratory spiked-in saliva assays (Fig. 3B). We tested the LFIA on human saliva samples from carriers with subclinical infection (as determined by microscopy) and demonstrated that the initial LOD for our LFIA strips was comparable to that of microscopy. Despite several replicate runs, we did not detect a false-positive signal using naïve human saliva spiked with a nontarget parasite protein, such as recombinant *P. falciparum* histidine-rich protein II (PfHRP2; ItG strain), or with a mixed trophozoite-schizont lysate. We tested the LFIA on human saliva samples from carriers with subclinical infection (as determined by microscopy) and demonstrated a comparable LOD. Our spiked assessment light microscopy (LM) estimates demonstrated an LOD range for the LFIA as ~1 to 16 gametocytes/µl of blood. This initial comparison was limited to those blood films where gametocytes and/or trophozoites were detectable by LM. The speed of the LFIA is dependent on the abundance of the biomarker in saliva, ranging from 3 to 5 min to as much as 30 min (Table 2). Drying the strips greatly improved the signal for samples that had low gametocyte density in the blood.

**Table 2:** Comparative microscopy, qPCR/PCR, LC-MRM, and LFIA for 100 samples collected from children with subclinical parasitemia from Cameroon. Time to readout for LFIA test line positivity and a maximum of 15 min for negative tests are included (right column).

Case code	Age	Microscopy		qPCR/PCR		PSSP17		
				Gametocyte	Trophozoite	Pfs25	18S rRNA	LC-MRM	LFIA	Time to readout
A115	12	-	-	+	+	0.05765	+	15
A120	11	-	+	+	+	0.03222	+	15
A187	8	-	+	+	+	0.01779	+	10
A193*	7	-	+	+	+	0.02164	+/-	15
A225	10	-	+	+	+	0.04005	+	>20
A227	11	-	+	+	+	0.02615	+	10
A268	10	-	+	+	+	0.04036	+	15
A270	10	-	+	+	+	0.04381	+	10
A278	11	-	+	+	+	0.01951	+	15
A279	12	-	+	+	+	0.02216	+	10
A290	10	-	+	+	+	0.05321	+	>20
A447	8	-	+	+	+	0.03030	+	15
A450	7	-	+	+	+	0.03537	+	>20
A452	10	-	+	+	+	0.01364	+	15
A460	8	-	+	+	+	0.03212	+	>20
A474	9	-	+	+	+	0.01039	++	5
A475	8	-	+	+	+	0.01483	+	10
A493	10	-	+	+	+	0.01932	++	5
B006	10	-	-	+	+	0.02097	+	10
B007	10	-	+	+	+	0.01784	+	>20
B023	9	+	+	+	+	0.05046	+	>20
B024	13	-	-	+	+	0.04245	++	5
B026	6	-	+	+	+	0.08123	+	15
B028	7	+	+	+	+	0.06404	++	5
B029	10	-	+	+	+	0.08174	+	15
B034	9	+	+	+	+	0.04904	+	5
B043	13	-	+	+	+	0.01549	+	10
B046	13	-	+	+	+	0.06638	+	10
B092	8	+	+	+	+	0.07328	+	5
B096	5	+	+	+	+	0.03484	+	15
B115	8	-	+	+	+	0.05615	+	10
B124	6	-	+	+	+	0.01231	-	15
B126	7	-	+	+	+	0.09663	+	15
B128	10	-	+	+	+	0.07039	++	5
B145	7	-	+	+	-	0.02850	++	5
B336	10	-	-	+	+	0.07140	++	5
B345	8	-	+	+	+	*0.00521*	++	5
B349	8	-	-	+	+	0.04474	+	10
B350	14	-	-	+	-	0.04074	+	15
B360	8	-	-	+	+	0.02313	-	15
B380	12	-	-	+	+	0.04243	++	5
B381	12	-	+	+	-	0.06924	-	15
B390	11	-	-	+	+	0.03683	+	10
B392	16	-	+	+	+	0.04583	+	15
C001	10	-	+	+	+	0.03759	+	10
C009	10	-	+	+	+	0.07004	+	5
C010	13	-	+	+	+	0.08945	++	5
C024*	12	-	+	+	+	0.06621	++	5
C026*	10	-	+	+	+	0.04700	+	15
C046*	12	-	+	+	+	0.02633	+	>20
C075	9	-	+	+	+	*0.01026*	+	10
C080	12	-	-	+	+	0.01808	++	5
C084*	8	-	-	+	+	0.04231	+	>20
C095*	10	-	-	+	+	0.01362	++	5
C100*	10	-	-	+	+	0.06038	+/-	10
C106	11	-	-	+	+	0.02973	-	15
C107	8	-	-	+	-	0.02309	+	10
C112*	10	-	-	+	+	0.01808	+/-	15
C117*	12	-	-	+	+	0.10120	+/-	15
C123	8	-	+	+	+	0.01950	++	5
C334	7	-	+	+	+	0.04649	+	5
C337	6	-	+	+	+	0.04951	+	10
C339	7	-	+	+	+	0.03356	+	15
C340	7	-	+	+	+	0.02086	+	10
C341	6	+	+	+	+	0.01425	+	>20
C357	5	-	-	+	+	0.05616	-	15
C358	8	-	-	+	+	0.02162	+	15
C363	8	-	+	+	+	0.04584	+	5
D030	11	-	+	+	+	0.03905	++	5
D033*	13	-	+	+	+	0.03652	++	5
D040	10	-	-	+	+	0.03692	++	5
D041	12	-	-	+	+	0.01479	-	15
D046	7	-	-	+	+	0.05459	+	5
D049	9	-	-	+	+	0.02713	+	>20
D053	12	-	-	+	+	0.04751	+	10
D054	6	-	+	+	+	0.01723	-	15
D057	8	-	-	+	+	0.02074	+	10
D062	9	-	+	+	-	*0.00967*	+	>20
D155	10	-	-	+	+	0.03353	+	15
D446	9	-	-	+	+	0.02517	+	10
D447	8	-	-	+	+	0.04891	+	15
D449	9	-	-	+	+	0.01917	+	>20
D453	9	-	+	+	+	0.04835	+	10
D456	9	-	+	+	+	0.01120	+	15
D459	13	-	+	+	+	0.04252	+	10
D461	13	-	-	+	+	0.02149	++	5
D462	11	-	-	+	+	0.03318	+	15
D482	10	-	+	+	+	0.03762	+	10
D487	9	-	+	+	+	*0.00445*	-	15
D496	7	-	-	+	+	0.02504	+	15
D499	9	-	-	+	-	0.06422	+	10
D500	8	-	-	+	-	0.01736	++	5
D516	8	-	-	+	+	0.03949	+	5
D517*	9	-	-	+	-	0.02459	++	5
N227	10	-	-	+	+	0.04180	++	5
N234	9	-	-	+	+	0.01287	+	10
N245	10	-	+	+	+	0.08426	+	10
N255	9	-	-	+	+	0.10392	+	15
N311	8	-	-	+	+	0.04298	+	10
N318	6	-	+	+	+	0.04264	+	10

* Samples included in Fig. 3D.

### Comparative profiling of PSSP17 LFIA, microscopy, LC-MRM, and PCR

We selected initially samples from Cameroon that were either microscopy negative/LC-MRM positive/qPCR positive or microscopy negative/LC-MRM negative/qPCR positive (Fig. 3C). Several LCMRM– positive samples, which were microscopy negative (A053, B357, B364, C007, C017, C020, C022, and C037), were positive by LFIA. Each one of these samples was found to be positive for gametocyte carriage by *pfs25* qPCR (table S5). We also noted that two replicate LC-MRM–negative samples (A048/A048b) that were analyzed independently to exclude potential experimental variability were positive by microscopy and qPCR. This is not surprising due to the extensive sample preparation that is needed for LC-MRM analysis, akin to what has been observed in the field for qPCR analyses. The LFIA was positive at the test line for both samples A048/A048b, demonstrating reproducible sampling from the same individual. Sample D492 was negative by both LC-MRM and microscopy, but was positive by LFIA and qPCR for *pfs25* (117 gametocytes/µl).

Considering the observed discrepancies in orthogonal detection approaches (Fig. 2D), we selected a second set of 10 samples where protein transcript discrepancies were obvious (Fig. 3D). Although most of the samples fell into one category (positive by smear for asexuals but negative for gametocytes, and positive by qPCR for asexuals and gametocytes), the selected samples represent the extreme outliers in our sample set. We further partitioned the LFIA detection data for all LC-MRM–positive saliva samples by microscopy (gametocyte versus trophozoite positivity) and PCR (pfs25 or 18S rRNA) results to identify instances where PSSP17 was detected by LFIA, but the sample was *pfs25* negative. Although LC-MRM was sufficiently sensitive in detecting PSSP17 in individual saliva samples, the LFIA readout (brightness of the test line signal) was not directly correlated with PARs. However, LFIA and LC-MRM positivity as a whole were in agreement. It is important to note that the LFIA prototype has not been fully optimized and the intrinsic differences in the detection approach do not immediately prescribe a direct correlation. We also observed that the LFIA can detect PSSP17 in the saliva of an individual with submicroscopic parasitemia (C084, C100, C112, and D517) and in individuals with no detectable gametocytes by microscopy (all samples). Several of the LFIA signals were weak (A193, C100, C112, and C117); despite the general agreement of LFIA and LC-MRM results, these samples point out issues with the prototype setup. For example, PSSP17 was present in case C100, with a PAR = 0.06038; this sample would have been predicted qualitatively to have a strong signal if compared to sample C024.

The analyses of 100 samples with matching microscopy (including the 10 in Fig. 3D), PCR, LC-MRM, and LFIA data are shown in Table 2. In this set of samples, we noted that some LC-MRM–negative samples (B345, C075, D062, and D487) showed disagreement between LFIA and LC-MRM data. B345, C075, and D062 were positive by LFIA, despite PAR estimates considered subjectively “negative”, due to the poor confidence measurement by LC-MRM. D487 is a case example where both LFIA and LC-MRM were negative despite molecular detection of gametocytes/trophozoites. Trophozoites were identified in the blood smear from D487 but not gametocytes, indicating that this individual is considered a clear, false negative. There were seven samples that were also found to be negative by LFIA: B124, B360, B381, C106, C357, D041, and D054. Of these, B124, B381, and D054 were positive for trophozoites by microscopy, indicating that these individuals would be otherwise missed using the current prototype PSSP17 LFIA. B360, C106, C357, and D041 were all submicroscopic (microscope negative) but PCR positive for both gametocytes and trophozoites, and again considered false negatives. B381 had a high PAR and was positive by microscopy for trophozoites but negative by PCR (18*S* rRNA). It is unclear why the LFIA failed to detect PSSP17 in this individual given the high PAR and general agreement between LC-MRM and the LFIA. Given the variability observed in terms of signal, we were not surprised to note that the estimated time to readout for each test also varied extensively. The LC-MRM did not predict either test line positivity or speed to read out on the LFIA.

**Fig. 4:**
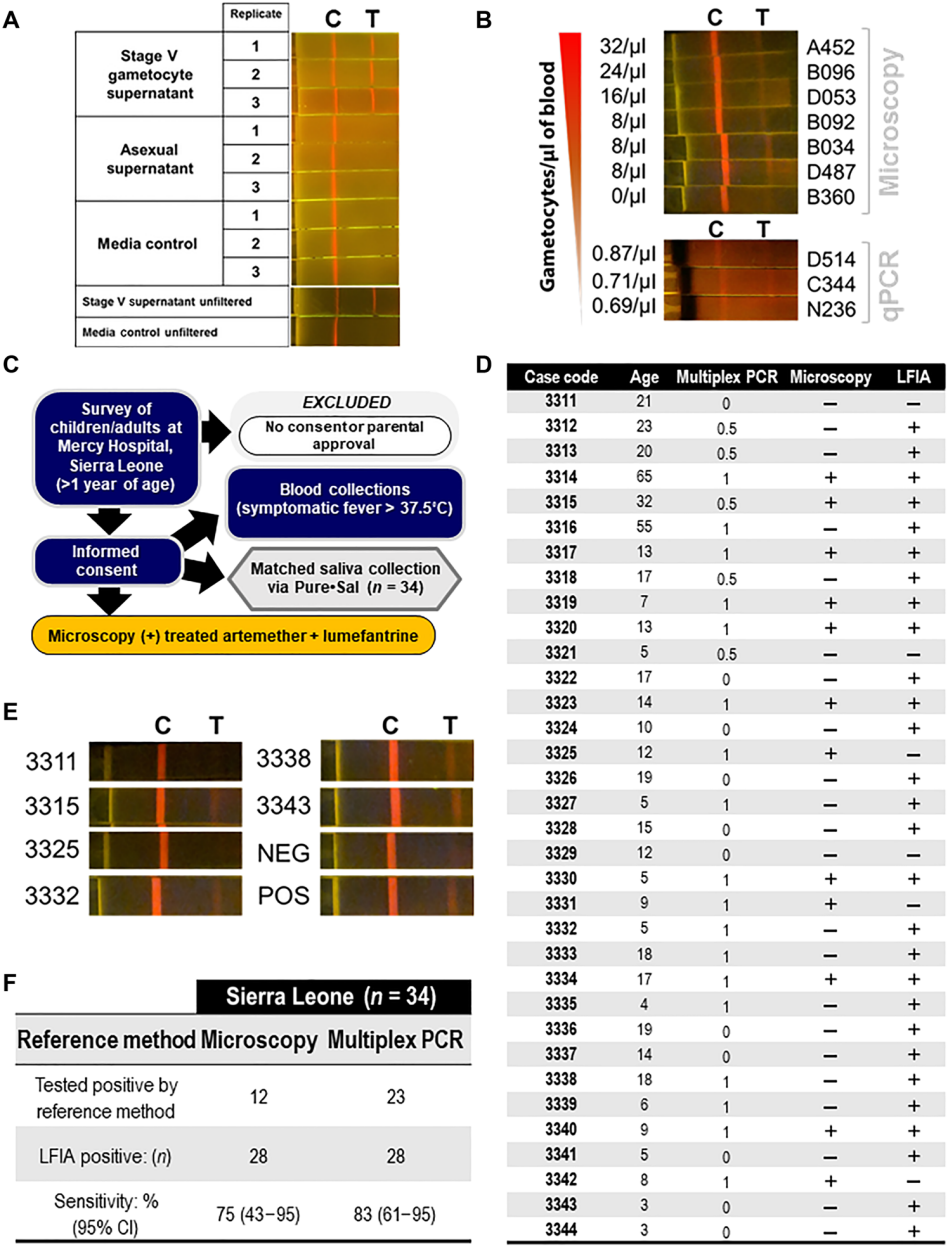
**Evaluation of the LOD of the prototype PSSP17 LFIA and potential use in clinical settings**. (**A**) Replicate tests using filtered (or unfiltered), axenic supernatant from stage V gametocyte cultures, asexual blood-stage cultures, and complete media (negative control). (**B**) Estimated LOD of the PSSP17 LFIA based on gametocyte quantification using LM or qPCR for *pfs25* transcripts. (**C**) Schematic of the Sierra Leone study to test the utility of the LFIA in confirmatory diagnosis of malaria parasite infection. Symptomatic individuals (>1 year of age) presenting in the Mercy Hospital in Bo, Sierra Leone, who provided informed consent were enrolled in the study. Matched blood samples were stored either in RNAlater or as DBS on Whatman FTA cards. (**D**) LFIA test strips showing PSSP17 detection in the saliva of a subset of symptomatic individuals presenting at a clinic in Bo, Sierra Leone. Negative control, uninfected/naive human saliva; positive control, naïve human saliva with lysed *P. falciparum gametocytes.* (**E**) Table comparing orthogonal detection approaches, multiplex PCR, LM, and the PSSP17 LFIA for all 34 samples from Sierra Leone. Multiplex PCR score: 0 = negative, 0.5 = weak positive, and 1 = positive. (**F**) Table estimating the sensitivity of the LFIA in a clinical setting compared to microscopy or multiplex PCR as the reference standard. For all lateral flow tests: C, control line; T, test line.

The discovery of PSSP17 in the saliva of children with subclinical parasitemias suggested that the marker is likely secreted into the GCF, because, to our knowledge, the presence of whole, intact gametocyte/trophozoites in saliva has never been reported. This is not surprising, given that saliva is a hypotonic solution. Using the LFIA, we determined whether PSSP17 is secreted into culture medium by mature, stage V *P. falciparum* gametocytes. We found that, in replicate tests, PSSP17 was detectable in axenic, filtered medium harvested from a day 18 gametocyte culture as compared to controls (filtered complete parasite culture medium) (Fig. 4A). Filtration assured that material from lysed stage V gametocytes would not contribute to a positive signal. As expected, axenic, filtered culture medium from asexual blood-stage culture, with both trophozoites and early schizonts, did not produce a positive signal because PSSP17 is gametocyte specific. Culture medium is changed daily in standard gametocyte culture protocols, and therefore, our data suggest that PSSP17 would have been secreted only during the preceding 24-hour period before harvest. Filtration itself is not necessary, and the data indicate that any material from lysed gametocytes did not result in an enhanced signal. The nature of the PSSP17 present in the culture medium remains unknown, but it is less likely to represent a single, soluble molecular species and may present as a surface-exposed protein on a lipid carrier or as a stabilized protein aggregate.

We had observed that samples from Cameroon that were positive for gametocytes/trophozoites by microscopy or molecular detection generally agreed with LFIA results (Fig. 3, C and D). In comparison with microscopy detection of gametocytes (the reference standard), the PSSP17 LFIA had an estimated sensitivity of 100% [95% confidence interval (CI), 59 to 100]; compared to microscopy detection of trophozoites, sensitivity was 92% (95% CI, 82 to 97). Considering molecular detection for *pfs25* or 18*S* rRNA as reference standards, the estimated test sensitivities are 92% (95% CI, 85 to 97) and 91% (95% CI, 84 to 96), respectively. Given that PSSP17 is primarily secreted by gametocytes, we estimated the LOD of the LFIA to be ~0.7 gametocytes/µl (Fig. 4B) blood equivalents, as determined by microscopy or quantitative reverse transcription PCR for *pfs25* transcript (table S5). These data suggest that our LFIA is potentially approaching the reported detection sensitivity of *pfs25* qPCR of about 0.02 gametocytes/µl (5), but in its current form, the prototype LFIA cannot reach this LOD. Here, we were limited in our comparative analysis: We are left with attempts to correlate estimated gametocyte densities in blood (*pfs25* qPCR) with the detection of a different gametocyte-specific protein marker in saliva (MRM and LFIA). The absolute quantification of PSSP17 in human saliva has not been performed to date, and such a study performed in the context of a comprehensive biomarker validation project is important in delineating the true LOD of this PON test.

Considering that gametocytes tend to be concomitantly present with asexual blood stages in symptomatic individuals and gametocytes derive from a preceding asexual blood-stage population, we had originally hypothesized that the LFIA should easily confirm *P. falciparum* infection. We tested this hypothesis by dovetailing on an ongoing study in a clinical setting in Bo, Sierra Leone. Saliva was collected from 34 individuals (ages 3 to 67) presenting at the Mercy Hospital in Bo (Fig. 4C). Because of the febrile nature of the individuals, the standard collection method used in Cameroon and Zambia was replaced with the use of the Pure•SAL saliva collection device, a simple “lollipop” instrument that rapidly and passively draws saliva within 3 min. We noted that there was a relatively strong concordance between the LFIA positivity and microscopy and PCR positivity (Fig. 4, D and E). The estimated sensitivity of the LFIA for symptomatic cases (Fig. 4F) with microscopy as the reference standard is 75% (95% CI, 43 to 95), and with multiplex PCR as the reference, it is 83% (95% CI, 61 to 95).

Two samples, case codes 3311 and 3329, which were negative by PCR and microscopy, were also negative by LFIA. However, we also observed several samples that showed discordant results between the LFIA and detection by either microscopy or molecular amplification. Case code 3321 was found to be positive by PCR, negative by microscopy, and negative by LFIA. Case codes 3325, 3331, and 3342 were found to be positive by both PCR and microscopy but negative by LFIA. Nine of the cases were found to be negative by PCR and microscopy but positive by LFIA. It should be noted that PCR detection of gene targets that are not specific to gametocyte stages alone would result in discordant results with the LFIA, especially among subclinical carriage (microscopy negative for either asexual or gametocyte stages).

## DISCUSSION

There is an unmet need for sensitive PON diagnostic tests that can identify subclinical carriage of *Plasmodium* asexual parasites and gametocytes in human populations in clinical, port, school, or home settings. Such a need is more pronounced in the context of malaria elimination and eradication, especially because countries entering the elimination phase will be faced with the challenge of detecting low-density parasite carriage in the midst of reductions in infection prevalence. Although there is a renewed focus on improving current blood-based tests that detect PfHRP2, because both gametocytes and asexuals have PfHRP2, and considering that at most only 2% of the entire parasite biomass are gametocytes (*27*), it is difficult to envision how PfHRP2 would be an appropriate biomarker for gametocytes. The data resulting from five rounds of RDT profiling tests that were conducted by the WHO in collaboration with FIND (Foundation for Innovative New Diagnostics) suggest that only a handful of existing blood-based RDTs can detect 200 asexual parasites/µl of blood. However, we have found that the lower limit of LM-detectable subclinical carriage ranges from 1 to 10 gametocytes/µl of blood. On the basis of our surveys of children in schools, subclinical carriage can occur with low numbers or (less frequently) the complete absence of asexual stages, more commonly occurring by mixed gametocyte/asexual infections, with densities 10-fold less than the lower limit tested in the WHO-FIND study. The major issue of the circulation of HRP2 deletion parasites in sub-Saharan Africa, which effectively limits the utility of HRP2 as a biomarker for malaria detection, cannot be ignored (12).

The potential utility of noninvasive biofluid sampling approaches for rapid malaria diagnosis has not been fully explored. Oral fluid offers an attractive option, given its inherently lower infection risk (absence of sharps during collection), cultural acceptance (as opposed to potential cultural blood taboos), and ample sample amount during a single collection (as opposed to 5 to 10 µl of blood sampled from finger pricks). In our study, 2 to 5 ml of saliva collections were performed quickly and could be performed independently by each subject, even for the 5-year-old children. Anecdotally, the children viewed the collection as a fun “spitting contest,” unlike the perception of finger-prick sample collection. However, recent efforts at identifying malaria parasite proteins in saliva have not been successful. A proteomic analysis of saliva from symptomatic individuals revealed only three proteins that achieved unequivocal protein assignments to *P. falciparum*: porphobilinogen deaminase (PF3D7_1209600) and two heat shock protein 70 isoforms, PfHSP70/PfHSP70-2 (PF3D7_0818900 and PF3D7_0917900) (28). The two HSP70 proteins, PF3D7_0818900 and PF3D7_0917900, with Mascot ions scores of 219 and 160, respectively, were also identified in our pooled saliva samples when we used an in-gel digestion followed by LC-MS/MS approach. Although they had been previously described to be present in saliva, due to the high degree of conservation of the sequence with human heat shock 70-kDa protein 1A/1B (P17066|HSP76_HUMAN), we excluded them from our final protein list. PfHRP2 has been detected previously in the saliva of malaria patients in Ghana (*29*) and the Philippines (*30*) by a sensitive sandwich ELISA (enzyme-linked immunosorbent assay) method, but to our knowledge, HRP2 has not been adequately measured in individuals with subclinical infection. PfHRP2 is a notoriously difficult protein to identify by MS and requires extensive sample enrichment. We trained and optimized our sample preparation and LC-MS/MS analysis methodology using in vitro cultured, mature stage V gametocytes (*23*), and in doing so realized that the previous efforts using lectin depletion methods (*28*) were unnecessary. These depletion methods may have led to sample loss as opposed to parasite protein enrichment. Our analysis of saliva from children with subclinical infection resulted in a 13-fold increase in the parasite protein repertoire of this biofluid. Given our experience with PfHRP2 and saliva proteomics, we do not anticipate the facile MS/MS detection of PfHRP2 in this biofluid. Rather, we found that ELISA was more effective in quantifying this protein.

We selected PSSP17 (PF3D7_1218800) as our candidate, female-specific gametocyte marker in this proof-of-concept study. The rationale for this selection was (i) PSSP17 was discovered in pooled saliva and individual saliva samples and (ii) PSSP17 was characterized as a female-specific gametocyte protein (*23*), therefore presumably more abundant in biofluid given the female to male gametocyte ratio of 4:1. LC-MRM offers an attractive strategy for profiling samples without the requirement for high sample concentration and low sample complexity, which usually hampers traditional MS approaches (*31*). MRM is neither a PON test nor a potential population-wide screening approach; however, LC-MRM did allow us to estimate the abundance of a candidate protein biomarker (*32*) and to evaluate its potential detection by antibody-based approaches (*33*), which are customarily used as the detection method for lateral flow-based tests such as malaria RDTs. We also noted an unanticipated retention time shift of 0.16 min for PSSP17 in our LC-MRM study, present in only the naturally infected saliva samples from children in Cameroon and Zambia. There are two likely possibilities responsible for this minimal shift, which was not observed when using “normal” naïve human saliva spiked with cultured stage V gametocytes. First, it is difficult to recapitulate the biology of children in malaria-endemic regions, which can include systemic conditions resulting from hemoglobinopathies, severe anemia, and other gastric conditions due to undernutrition. Such conditions can lead to increases in bile acids, iron concentrations, and other saliva matrix interferences that can affect retention times (*34*). However, we successfully confirmed the presence of gametocytes in a subset of these children by qPCR, when only the LC-MRM analysis indicated positive carriage, demonstrating the presence of stage V gametocytes in the blood. Furthermore, we used the LFIA to demonstrate orthogonally that protein was present in the saliva of these children. The true LOD, sensitivity, and specificity for the PON test based on the PSSP17 marker in saliva have not yet been determined and are the focus of the next stage in the preclinical assessment and field validation of this noninvasive RDT using absolute quantitative proteomic analyses.

A major strength of our study is that all LC-MRM analyses were performed on blinded samples, where the LM and qPCR data were unknown. Furthermore, by selecting random samples with paired datasets (qPCR and LC-MRM), we reduced the bias that would otherwise disproportionately influence how well the lateral flow rapid test performed. Taking this approach allowed for subsequent rechecking of individual samples with matched orthogonal measurements to assess the limits of detection of the LFIA. A limitation of this study is our reliance on *pfs25* qPCR assays to estimate gametocyte density in blood, which is a different biofluid than that used in the LFIA. The sampling of finger-prick blood (100 µl) from individuals with very low gametocyte densities (A042 and A081) can lead to a failure to amplify *pfs25*, presumably due to the relatively poor chance of sampling a gametocyte; however, our lateral flow test detected PSSP17 in 10 µl of saliva from these two children. We anticipate that additional development is needed where we can leverage our known standard concentrations of PSSP17 (detectable by lateral flow) and their correlation with gametocyte densities. However, because our hypothesized mechanism relies on a secreted PSSP17 marker, such studies will need to use axenic culture medium, as opposed to serial dilutions of purified, gametocyte-infected red cells. Another important consideration is the potential temporal relationship between the detection of PSSP17 in saliva with gametocyte and trophozoite presence in the blood. Akin to the known stable presence of HRP2 in blood, which represents an infection that is being cleared, PSSP17 may remain in saliva even though gametocytes have been cleared in blood. This may be one explanation for the noted discrepancies between molecular detection of gametocytes/asexuals in blood and the positive LFIA signal indicating the presence of PSSP17 in saliva. However, a particularly provocative notion would be that mature gametocytes that sequester in capillary beds—or even developing gametocytes, sequestered in bone marrow—may secrete PSSP17, resulting in the failure to detect gametocytes by microscopy or PCR. To assess utility of the LFIA test for epidemiological landscaping, further evaluation will need to be performed in areas where malaria transmission has been reduced so drastically that it is low and unstable.

One of the limitations we noted during the progressive development of the LFIA was that, depending on lot/batch number of the chromatographic strip, the detection antibody (mAb 27C9.B5-EuChelate particle conjugate) can become partly trapped in the loading pad–membrane interface. This leads to a reduction of a positive signal in the test line, a control line, or both. This variability in the manufacturing of the lateral flow strip itself did not necessarily invalidate the interpretation of the presence/absence of PSSP17 for a given saliva sample. In our study, for a test to be considered positive, both the test and control line must have a positive signal (regardless of intensity). The prototype test is not quantitative in its current form, so variation in signal intensity does not have any implied significance (no correlation with parasitemia). A positive signal on a validated diagnostic test, albeit faint, would still trigger the same response (treatment). We expect that this potential issue could be resolved during development and optimization of a commercial test kit using this technology.

The potential of using the same rapid test for detecting *Plasmodium vivax* subclinical gametocytemic carriage, which is concurrent with asexual parasite infection, compels further study. The vivax ortholog of PSSP17 shares >80% sequence identity at the amino acid level. A single noninvasive PON RDT for both human malaria parasites would surpass the WHO recommended response to the serious public health concern of the increased reporting of *pfhrp2/pfhrp3* deletion mutants in sub-Saharan Africa and South America (*12*). Although our analyses of saliva from 34 symptomatic individuals indicate a potential use of the prototype LFIA in the clinical management of malaria, our primary envisioned application is implementation for epidemiological studies that necessarily go beyond the confines of a clinical setting. Again, by targeting a gametocyte stage protein, we hope to identify individuals with subclinical infection with co-circulating asexual blood stages and mosquito-infectious gametocytes, which, in turn, can help map transmission hotspots more accurately in regions with heterogeneous malaria transmission. Our identification of other parasite-derived antigens in the saliva such as PF3D7_0507800 and PF3D7_0906100, both of which are shared between gametocytes and asexual stages, can lead the way to an optimized, highly sensitive saliva-based RDT for both research and clinical settings. Taking this notion a step further, we propose that pairing such a diagnostic with “stamp out” interventions such as a malaria transmission–blocking vaccine would permit a more targeted immunization strategy that may lower the cost of such campaigns.

## MATERIALS AND METHODS

### Study design

The overall goal of the project is to leverage MS-based proteomic approaches to discover and quantify *P. falciparum* protein markers present in human saliva and explore the possibility that one parasite protein marker, in particular PSSP17, can be detected via a noninvasive, prototype LFIA. We mined the saliva of children with subclinical malaria parasite infection and down-selected from the catalog of proteins identified to a single, female gametocyte-specific candidate for this proof-of-concept study. A competitive profiling, cross-sectional, multi-omics study of unstimulated saliva and matched blood samples from 364 subclinical malaria parasite infection in Cameroon (in primary schools) and Zambia (in households) was completed to evaluate the utility of this target saliva-based marker in quantifying the subclinical population. To expand the potential utility of the prototype LFIA test, saliva was collected from children and adults (with symptomatic fever >37.5°C) presenting at a clinic in Sierra Leone. Informed consent was acquired for each of the 399 biofluid samples collected from the three different countries representing West, Central, and Southern Africa. These independent collections were nested with ongoing research studies and community surveys, and followed National Malaria Control Program guidelines for referral of microscopy-positive individuals for appropriate treatment. These protocols have been approved by the relevant institutional review boards and national ethics committees for Cameroon, Zambia, and Sierra Leone (2015/07/613/CE/CNERSH/ SP; TDRC/ERC/2010/14/11/IRB#00003467; IRB#477605-6/ IRB#NRL.2012.0007).

### Statistical analysis

The Mascot ions score significance threshold [−10Log(*P*)] reported in Table 1 and table S1 is based on the probability, *P*, that the observed “match” between the experimental data and the corresponding database sequence pulled from the combined database search is random. Tests for correlation and subsequent figure generation were conducted in program R (*35*). Calculation of sensitivity of the LC-MRM and LFIA tests was done using the *pfs25* qPCR and 18*S* rRNA PCR as reference method for Cameroon and Sierra Leone, and the *cytB* nested PCR as reference method for Zambia. Binomial confidence interval was calculated by “cii” (confidence interval immediate) command specifying “proportions” using STATA version 14 (StataCorp LP).

## SUPPLEMENTARY MATERIALS

www.sciencetranslationalmedicine.org/cgi/content/full/11/473/eaan4479/DC1

Materials and Methods

Fig. S1. The correlation of PAR and female gametocyte abundance per microliter of blood (based on *pfs25* transcript number).

Table S1. The complete list of *P. falciparum* proteins identified in the saliva from children with subclinical infection in Yaoundé, Cameroon.

Table S2. LM analyses of blood samples from children (5 to 12 years old) with subclinical infections in Yaoundé, Cameroon.

Table S3. Description of samples collected from schools in Cameroon.

Table S4. Description of samples collected from homes in Zambia.

Table S5. Quantification of gametocytes per µl of blood in a subset (n = 100) of samples from Cameroon.

References (36–44)

## Supplementary Material

A saliva-based rapid test to quantify the infectious subclinical malaria parasite reservoir

## Data and materials availability

All processed data associated with this study are present in the paper or in the Supplementary Materials. Raw MS data, used in the current study (PASS01290), have been deposited in Peptide Atlas and can be accessed through this link: (http://www.peptideatlas. org/PASS/PASS01290).
