# Supplementary material for: A saliva-based rapid test to quantify the infectious subclinical malaria parasite reservoir
Source: Sci Transl Med. 2019 Jan 2;11(473):eaan4479. doi: 10.1126/scitranslmed.aan4479 (PMC6441545; doi:10.1126/scitranslmed.aan4479)
Supplement: A saliva-based rapid test to quantify the infectious subclinical malaria parasite reservoir [file STM-11-eaan4479-s001.pdf]

Science Translational Medicine
AAAS

www.sciencetranslationalmedicine.org/cgi/content/full/11/473/eaan4479/DC1

Supplementary Materials for

A saliva-based rapid test to quantify the infectious subclinical malaria parasite reservoir

Dingyin Tao, Brent McGill, Timothy Hamerly, Tamaki Kobayashi, Prachi Khare, Amanda Dziedzic, Tomasz Leski, Andrew Holtz, Bruce Shull, Anne E. Jedlicka, Andrew Walzer, Paul D. Slowey, Christopher C. Slowey, Sandrine E. Nsango, David A. Stenger, Mike Chaponda, Modest Mulenga, Kathryn H. Jacobsen, David J. Sullivan, Sadie J. Ryan, Rashid Ansumana, William J. Moss, Isabelle Morlais, Rhoel R. Dinglasan*

*Corresponding author. Email: rdinglasan@epi.ufl.edu

Published 2 January 2019, *Sci. Transl. Med.* **11**, eaan4479 (2019)
DOI: 10.1126/scitranslmed.aan4479

The PDF file includes:

Materials and Methods
Fig. S1. The correlation of PAR and female gametocyte abundance per microliter of blood (based on *pfs25* transcript number).
Table S1. The complete list of *P. falciparum* proteins identified in the saliva from children with subclinical infection in Yaoundé, Cameroon.
Table S2. LM analyses of blood samples from children (5 to 12 years old) with subclinical infections in Yaoundé, Cameroon.
Legends for tables S3 to S5
References (*36–44*)

Other Supplementary Material for this manuscript includes the following:

(available at www.sciencetranslationalmedicine.org/cgi/content/full/11/473/eaan4479/DC1)

Table S3 (Microsoft Excel format). Description of samples collected from schools in Cameroon.
Table S4 (Microsoft Excel format). Description of samples collected from homes in Zambia.
Table S5 (Microsoft Excel format). Quantification of gametocytes per µl of blood in a subset (*n* = 100) of samples from Cameroon.

Supplemental Materials

Materials and Methods

Mass spectrometry analyses

Saliva protein digestion

The saliva samples were filtered through a 0.22 μm filter (Thermo Fisher Scientific) to remove debris and then buffer-exchanged into 50 mM ammonium bicarbonate using Amicon Ultra 0.5 mL 3K centrifugal filters (EMD Millipore). Protein concentration was determined by Bicinchoninic Acid Assay (BCA) using 96-well plate. Protein (about 50 μg) from each sample was used for in-solution digestion, followed by denature, reduction, alkylation and incubated with trypsin (1:50, trypsin/protein) at 37 ^{0}C for around 12 hours. Acidified tryptic peptides were desalted using an High Performance Liquid Chromatography (HPLC) C18 column on an Agilent 1200 HPLC system (Agilent Technologies), lyophilized and then re-suspended in 2% acetonitrile (ACN), 97.9% water and 0.1% formic acid (FA) buffer for liquid chromatography tandem mass spectrometry (LC-MS/MS) analysis.

LC-MS/MS for saliva protein identification

Digested peptides (about 20 μg) were injected to our constructed online 2-D HPLC-MS/MS system, using a method described previously (*23*). In brief, to construct the online 2D method, we integrated one strong cation-exchange (SCX) column (150 μm internal diameter (i.d.)* 2 cm length, PolySULFOETHYL ATM, 5 μm 300 Å, PolyLC, Inc.) into an Agilent LC-MS system comprised of a 1200 LC system coupled to a 6520 Quadrupole Time of Flight (QTOF) via a HPLC Chip Cube interface. For the online SCX fractionation, in the first dimension peptides were loaded into the SCX column at 1.8 μL/min and the peptides were eluted using the autosampler by injecting 6 μL of each increasing salt concentration (0, 15, 30, 45, 60, 120, 160, and 300 mM NaCl in 2% ACN/0.1% FA; followed by one injection of 500 mM NaCl in 2% ACN/0.1% FA to wash the column). The salt elution was captured by a C18 enrichment column integrated in the Agilent Polaris-HR-Chip-3C18 chip (360 nL, 180 Å C18 trap with a 75 μm i.d., 150 mm length, 180 Å C18 analytical column). In the second dimension, with valve switched

and the reversed phase liquid chromatography gradient started, the peptides were eluted from the enrichment column and separated by a C18 analytical column. Elution of peptides from the analytical column was performed using a gradient starting at 97% A (A: 99.9% water, 0.1% FA) at 300 nL/min. The mobile phase was 3–10% B for 4 min, 10–35% B for 56 min, 35–99% for 2 min, and maintained at 99% B (B: 90% ACN, 9.9% water, 0.1% FA) for 6 min, followed by re-equilibration of column with 3% B for 10 min. Data dependent (autoMS2) MS acquisition was performed by an Agilent 6520 QTOF at 2 GHz. Precursor MS spectra were acquired from *m/z* 315 to 1700 and the top 4 peaks were selected for MS/MS analysis. Product scans were acquired from *m/z* 50 to 1,700 at a scan rate of 1.5 spectra per second. A medium isolation width (∼4 amu) was used, and a collision energy of slope 3.6 V/100 Da with a 2.9 V offset was applied for fragmentation. A dynamic exclusion list was applied with precursors excluded for 0.50 min after two MS/MS spectrum was acquired.

Liquid Chromatography Multiple Reaction Monitoring (LC-MRM) quantification

An Agilent 1290 Infinity UHPLC system was used to directly inject 10 µL of desalted digest samples spiked with 100 fmol C13 heavy labeled internal standard peptide IQESSPAILINTK (6C13) (∼10 µg) onto a reversed phase analytical column (150 mm × 2.1 mm i.d., Agilent Poroshell 120 EC-C18, 2.7 µm particle size) that was maintained at a column temperature of 40 ^{0}C. Elution of peptides from the analytical column was performed using a gradient starting at 97% A (A: 99.9% water, 0.1% FA) at 0.5 mL/min. The mobile phase was 3-10% B for 3 min, 10-45% B for 9 min, 45-99% for 1 min, and maintained at 99% B (B: 90% ACN, 9.9% water, 0.1% FA) for 3 min, followed by re-equilibration of column with 3% B for 4 min. An Agilent 6490 Triple Quadrupole (QQQ) LC/MS with iFunnel technology, controlled by Agilent's MassHunter Workstation software (version B.06.00), was used for all LC-MRM/MS sample analyses. All acquisition methods used the following parameters: 3000 V capillary voltage and a 1500 V nozzle voltage, a sheath gas flow of 11 L/min (nitrogen) at a temperature of 250 ^{0}C, a drying gas flow of 14 L/min at a temperature of 200 ^{0}C, nebulizer gas flow at 20 psi, collision energy set at 29 V, Q1 set to unit resolution (0.7 FWHM) and Q3 set to wide resolution

(1.3 FWHM). MRM acquisition methods were composed of three ion pairs during the determination of high-signal producing interference-free transitions and LC method development. A default 380 V fragmenting voltage and 5 V cell accelerator potential were used for all MRM ion pairs, and the dynamic MRM option was used for all data acquisition with a target cycle time of 500 ms and delta EMV at 200.

Database searching and LC-MRM quantitation

All the LC-MS/MS raw data were converted to Mascot generic format (.mgf) by Agilent MassHunter Qualitative Analysis B.04.00. Mascot version 2.4.1 was used to search a combined database of SwissProt Human and *Plasmodium falciparum* sequences from GeneDB (2013.02), which consists of 28,960 entries for peptide sequence assignments using the following parameters: precursor ion mass tolerance of 50 ppm and a fragment ion mass tolerance of 0.2 daltons. Peptides were searched using fully tryptic cleavage constraints, and up to two internal cleavage sites were allowed for tryptic digestion. Fixed modifications consisted of carbamidomethylation of cysteine. Variable modifications that were considered were oxidation of methionine residues. Mascot's ions score significance threshold [-10Log(*P*)] reported in **Table 1** and **table S1** is based on the probability, *P*, that the observed "match" between the experimental data and the corresponding database sequence pulled from the combined database search, is in fact random.

Three MRM transitions for the peptide consist of two pair of y ions transitions (y8, 710.4->875.5, 707.4->869.5 and y5, 710.4->594.3, 707.4->588.4), which has a 6-Da difference between internal heavy labelled standard peptide and saliva peptide on the product ions, and one pair of b ions (b2, 710.4->242.2, 707.4->242.2), which should have the same product ions. The integrated Peak Area Ratio (PAR), which was calculated using saliva sample divided by heavy labeled internal standard through Agilent MassHunter Quantitative Analysis B.04.00 was used for the saliva sample quantitative comparison across all the samples.

Cameroon biofluid collection and survey

Ethical statement

All procedures for human sample collections were approved by the Cameroonian national ethical committee (2015/07/613/CE/CNERSH/SP).

Study Site

Sample collections were performed among children, 5 to 16 years old, attending primary schools in the Mfou district, a small town located 30 km southeast of the capital Yaoundé in Cameroon. The study sites (schools) are within a forest area located in the central region of Cameroon (**Fig. 1)**. Two de-identified, archived (frozen) saliva samples from a different IRB-approved study from individuals presenting with malaria in the Centre de Sante Catholique de NLONG-KAK clinic in Yaoundé were also used.

Study design

Volunteers were enrolled after obtaining their parent's or legal guardian's signed informed consent forms. Saliva samples were collected in OMNIgene collection tubes (DNA Genotek). Before collections, participants were screened for oral health problems or injuries, since the inclusion of blood in the saliva samples confounds analysis. None of the children included in our study were excluded based on this screen. Children were invited to rinse their mouth with 5 mL water to remove food residues. Advice was provided to help in oral fluid collection: thinking about favorite foods, making chewing movements or caressing their teeth with their tongue. Participants were asked not to spit, to avoid bubbles and ensure proper fluid collection. Donors delivered 2 mL of oral fluid and these saliva samples were collected within 10 minutes. Saliva samples visibly contaminated with blood were discarded. A 200 µL volume of Protease Inhibitor Cocktail (1:100 dilution, Roche) was added to the saliva samples, the mixture was vortexed and then completed with 500 µL 100% ethanol. Samples were stored at -20°C until processing.

Matched-blood samples were collected by finger prick. A 100 µL volume of blood was pipetted and dispensed into a 1.5 mL Eppendorf tube containing 250 µL of RNAlater and the tubes were kept at -20°C. Thick blood smears were calibrated to 10 µL, stained with a 10% Giemsa solution and examined under a Leica microscope (x100 magnification). Gametocyte density was estimated by counting parasite number against 1000 white blood cells (WBCs) and density of asexual stages against 500 WBC. Parasite numbers were converted to parasites/µL assuming the standard number of 8,000 WBCs per µL of blood. Children with asexual parasitemia exceeding 50 parasites/µL were treated with dihydroartemisin–piperaquine (Malacur) according to national guidelines.

Zambia biofluid collection and survey

Ethical Statement

The study was approved by the Institutional Review Boards of the Tropical Diseases Research Centre in Zambia (TDRC/ERC/2010/14/11) and the Johns Hopkins Bloomberg School of Public Health (IRB #00003467).

Study Site

Saliva samples were collected from residents of Nchelenge District, Luapula Province, Zambia in October 2013. Nchelenge District is located on the south eastern shore of Lake Mweru on the border with the Democratic Republic of Congo. The marshland environment provides breeding sites for *Anopheles funestus*, *An. gambiae* and *An. arabiensis* (*36*–*38*). According to the 2012 Zambia National Malaria Indicator Survey, the prevalence of RDT positivity among children younger than 5 years of age in Luapula Province was 56%, the highest parasite prevalence in Zambia (*39*).

Study design

The saliva collection was nested within ongoing malaria community surveys conducted by the Tropical Diseases Research Centre (TDRC) as part of the Southern Africa International

Centers of Excellence for Malaria Research (ICEMR). Satellite images were used to construct a sampling frame for the random selection of households as previously described (*37*). Briefly, the sampling frame was established using a Quickbird satellite image obtained from Digital Globe Services, Inc. The image was imported into ArcGIS 9.2 and locations of households were identified and enumerated manually. Structures of appropriate size and shape were identified as potential residences. After obtaining permission from the head of household and individual written informed consent, a questionnaire was administered to each participant older than 16 years of age residing within the household and to parents or guardians of those younger than 16 years of age.

A blood sample was collected by finger prick for a malaria rapid diagnostic test (RDT) and preparation of a dried blood spot (DBS). The DBS was collected on filter paper (Whatman, Protein Saver card 903), dried overnight and stored individually with desiccant in a sealed plastic bag at -20°C (*40*). The SD Bioline Malaria Ag Pf RDT (FIND Diagnostics) was used to detect *P. falciparum* histidine-rich protein 2 and individuals who were RDT positive were offered treatment with artemether-lumefantrine (Coartem). For oral fluid collection, individuals younger than 16 years of age were enrolled. Participants were informed not to eat at least 2 hours prior to the sample collection and were invited to rinse mouth with 5 mL water to remove food residues. Participants were asked to provide 2 mL of saliva in a sterile tube (DNAgenotek) containing one quarter of protease inhibitor cocktail (1:100 PIC, Roche). To stabilize the proteins, 500 μL of ethanol was added to the saliva and PIC mix to achieve a 20% (v/v) of ethanol. The saliva samples were kept in the cooler with ice packs during the field collection and were stored in the freezer at the field station. Samples were then transported to the US with ice packs.

Sierra Leone biofluid collection and survey

Ethical statement

All procedures for human sample collections were approved by the Sierra Leone Ethics and Scientific Review Committee, George Mason University IRB (#477605-6) and Naval

Research Laboratory IRB (#NRL.2012.0007) as part of the project titled, “Tiered Laboratory Analysis for Common Infections to Characterize Febrile Morbidity not Related to Malaria” in Sierra Leone.

Study Site

Sample collections were performed among febrile subjects reporting to Mercy Hospital, a small private hospital located in Kulanda Town section of Bo, the second largest city in Sierra Leone and capital of Southern Province. Mercy Hospital refers approximately 2000 patients to its own clinical lab for testing with *P. falciparum* malaria being the most prevalent pathogen detected in 20%-30% of tested individuals. Since the assays used in the study have not yet received approval for human diagnostic use, no clinical decisions were based on the results of the tests. Symptomatic individuals received standard-of-care infectious disease diagnostic testing free of charge, which in this case included malaria smear and/or Standard Diagnostics Bioline Pf/Pan RDT, along with standard medical care and treatment in Mercy Hospital.

Study design

The subjects were invited to the study if they were febrile [>37.5°C (oral) or >38°C (axillary)] at the time of the enrollment or experienced a self-reported fever in up to 7 days earlier. Individuals aged 3 and up qualified for enrollment after giving informed consent according to the procedures in the approved IRB protocols. Parental consent was required for subjects younger than 18 years old.

Sample collection, storage, and transport

Samples were collected between June 22 and July 06, 2017 (ages [3-4]=3, [5-8]=8, [9-12]=5, [13-16]=5, [17-20]=8, [21+]=6). After patient consent was received, a questionnaire was completed to collect information on previous infections, household, demographics, and antibiotic usage. Whole blood was collected using EDTA vacutainer tubes (Becton Dickinson) and stored in 1.5 mL screw cap microfuge tubes at -20°C. Additional 100 µL aliquots were stored in

RNAlater at -20°C and 50 µL aliquots were spotted onto Whatman FTA cards. Considering that individuals presenting in the clinic are febrile and the collection methods used previously for individuals with subclinical infection were deemed to be cumbersome, we used the Pure•SAL Saliva Collection Device (Oasis Diagnostics), which acquires a highly purified saliva specimen collected in standardized fashion in an average of about 1-3 minutes. Specimens are collected by means of an "inert" absorbent pad placed in the pool of saliva that gathers in the oral cavity. Collection is continued until a Sample Volume Adequacy Indicator [SVAI] built into the Pure•SAL device changes color from white to bright red signifying sufficient saliva has been collected for processing. The device collects between as much as 1.0 and 1.2 mL of a highly purified, non-viscous fluid that has been used without further purification in certain downstream technologies. The samples collected using the Pure•SAL Saliva Collection Device are 97% cell free and contain none of the interfering factors [mucins, other aggregation factors] that can cause downstream interference with research or clinical tests. In preliminary tests using the collection device, we had confirmed that the PSSP17 marker was not selectively excluded as a result of using this collection device. In our use in Sierra Leone, approximately 700 -1000 µL of filtered saliva was collected from each individual. To stabilize the samples, 100 µL of 1:10 dilution PBS and 200 µL of 90% ethanol was added to the saliva and stored at -20°C. Whatman FTA cards, extracted DNA, saliva, and blood samples were then triple sealed in specimen biohazard transport bags and transported to the U.S.

Cytochrome B (*cytB*) nested PCR detection

To detect submicroscopic malaria parasites in samples from Zambia, DNA was extracted from DBS using the chelex boiling method as previously described (*9, 38, 40*). A 50 µL blood spot was added to a 1.5 mL tube, red blood cells were lysed with 0.1% saponin/PBS, and 2% chelex/PBS was added, following which the tube was boiled for 8 minutes. The sample was centrifuged for 1 minute at 14,000 rpm and stored at minus 20°C or processed for PCR immediately. A nested PCR targeting the mitochondrial *cytB* gene was performed to detect the presence of human *Plasmodium* species using previously described primers (*9*).

Gametocyte *pfs25* qPCR

RNA Extraction, Reverse Transcription, and qPCR

For matched blood samples from Cameroon, RNA was extracted from 100 µL of blood stored in 250 µL of RNAlater using Ambion's RiboPure Blood kit according to manufacturer's instructions. RNA quantitation was performed using a NanoDrop spectrophotometer and quality assessment was determined by RNA Nano LabChip analysis on an Agilent BioAnalyzer 2100. cDNA was generated from 100 ng of RNA using Life Technologies SuperScript III First Stand Synthesis System. Quantitative PCR was performed with 1:5 dilution of cDNA in a 25 µL reaction in a Step One Plus machine using Life Technologies SYBR Green PCR Master mix with primers at 400 nM concentration. Reaction conditions: 95°C at 10 mins for 1 cycle, 95°C at 15 sec and 60°C at 1 min for 40 cycles, followed by standard melt curve analysis. The gene of interest was 25 kDa ookinete surface antigen precursor (*pfs25*) and the standard curve was generated using cDNA from a gametocyte culture. Each cDNA sample was run in triplicate, and the "no template" controls remained negative. The calculation for the detection threshold is provided in **table S5**. The range in the total number of gametocytes/µL of blood is 0.12 to 6,985 gametocytes/µL of blood.

18s ribosomal qPCR

To determine the number of individuals with subclinical infection carrying either asexual or sexual stages, the RNA was extracted from the available RNAlater-stabilized blood samples (*n* =244) from Cameroon using the TRIzol extraction protocol followed by purification using the RNeasy kit (Qiagen). The concentration of the RNA eluted in RNase free/ DNase free water was determined using the Nanodrop 2000 spectrophotometer (Thermo Fisher Scientific). As the yield from the field samples was very low, 11 µL of the RNA template was used for cDNA synthesis. The forward primer (5'-AAGTTAAGGGAGTGAAGA-3') was obtained from Billman, et al., (*41*) and the reverse primer (5'-CGTTCGTTATCGGAATTA-3') was designed and tested prior to being used for reverse transcription of 18S rRNA and qPCR analysis of the samples.

SuperScript ®IV Reverse transcriptase kit (Invitrogen Life technologies) protocol was used for the reverse transcription of the RNA. Standard SYBR-Green based qPCR protocol was followed to determine the presence of the 18S ribosomal RNA in the reverse transcribed samples. The qPCR reactions were performed using an Illumina Eco qPCR device and the thermal cycling was performed as follows: 10 mins at 95°C, followed by 40 cycles of 95°C for 10 sec, 45°C for 30 sec, 95°C for 5 mins. A melt curve was generated at the end of each reaction by cycling the temperatures as follows: 45°C-60 sec and 95°C- for enzyme deactivation. Positive control (RNA extracted from *in vitro P. falciparum* NF54 cultures and reverse transcribed to cDNA) and negative controls (no-reverse transcriptase control) were run alongside each sample set.

Multiplex qPCR malaria parasite detection

DNA extraction was done using the Qiagen DNA Blood Mini Kit (Qiagen). DNA Concentration was determined using the NanoDrop spectrophotometer (ABI/Thermo Fisher). The multiplex malaria assay used was developed by Walter Reed Army Institute of Research (WRAIR) (*42*). The multiplex malaria assay uses TaqMan chemistry and contains four primer/probe sets for detection *Plasmodium* spp., *P. falciparum* and *P. vivax* 18S rRNA targets and human RNaseP gene (control target). All samples with visible amplification of *P. falciparum* or *P. vivax* targets as determined by analyzing amplification curves were deemed malaria positive. Samples with C_t values >35 were classified as weakly positive. The universal malaria assay was based on the method developed by Mangold (*43).* Consensus primers were used to amplify 18S rRNA gene, and SYBR Green chemistry was used for amplicon detection. The target detection and species determination was based on melt curve analysis and calibrated using *P. falciparum* genomic DNA. The samples with melt curve peak at 75˚C were considered positive for *P. falciparum*.

Lateral Flow Immunoassay (LFIA)

Monoclonal Antibody Production

Five hybridoma clones were produced (GenScript) and supernatants were screened to

identify the optimal pair of mAbs for capture and detection of PSSP17 (mAbs 27C9.B5 and 10E2.B7). These five hybridomas were down-selected from the pool of hybridomas at every step of the process following cell fusion screening and subcloning using gametocyte lysates as antigen for indirect and sandwich ELISAs as well as western blots. Asexual parasite lysates were used as negative control (*23*). Epitope binning was performed to identify mAb antibody pairs. Affinity measurements via Biacore T200 were performed by GenScript to further down-select to the antibody pair described herein and in PCT/US2016/037968.

Antibody Conjugation

Anti-PSSP17 (PF3D7_1218800) detection mAb 27C9.B5 IgG was covalently coupled to carboxylate-modified polystyrene microparticles (MPs, 1% w/v) with 0.099 µm diameter, 37 $Å^2$ parking area, and Europium (III)-chelate (Thermo Fisher Scientific). 1-ethyl-3-(3-dimethylaminopropyl) carbodiimide hydrochloride (EDC, Thermo Fisher Scientific) was diluted to 10 mg/mL. Coupling reactions were performed using 200 mM and 20 mM sodium tetraborate decahydrate buffer (pH 9), as well as 25 mM MES buffer (pH 6.1). A 3% solution of methoxypolyethylene glycol amine (PEG, Mn=5000 g/mol, Sigma Aldrich) in 20 mM borate buffer was used for blocking MPs.

Antibody immobilization procedure was adapted from previously published methods (*26, 44*). To activate MP carboxyl groups, 30 µL MPs in 120 µL MES buffer was combined with 15 µL EDC to achieve an EDC:COOH stoichiometric ratio of 1. The reaction was incubated for 20 min and then 125 µL borate buffer (200 mM) and 23 µL of 27C9.B5 (941 µg/mL) were added to achieve 1.354 mg Ab/m^2 of MP surface area and incubated for 1 h. MPs were pelleted by centrifugation at 10,000 x g for 25 min, the supernatant was discarded and the pellet was resuspended via sonication in 300 µL borate buffer (20 mM). PEG solution was added to a concentration of 0.1% (w/v) and the reactions was incubated for 30 min, centrifuged, and resuspended in borate buffer (20 mM) as before and stored at +4 °C for at least 24 hours before use. Final MP-27C9.B5 conjugate (MPAb) concentration was 0.1% (w/v). The anti-PSSP17 capture mAb 10E2.B7 IgG (Bio-Ab) was biotinylated using EZ-Link NHS-PEG4-Biotinylation Kit

(Thermo Fisher) at a 20:1 Biotin: Antibody molar ratio.

PSSP17 marker capture and detection using the LFIA strip

Sample Dilution Buffer (SDB50), Matrix Reduction Buffer (MRB), and generic rapid assay device (gRAD) OneDetection strips were provided by BioPorto). Incubation steps were carried out at room temperature (RT) with gentle shaking. Assays were carried out by combining 10 µL of MRB:SDB50 (MRB final concentration 0.1% v/v) with either 10 µL of human saliva samples or control naïve human saliva spiked with recombinant PSSP17 to concentrations of 100 ng/mL to 0.5 ng/mL. To eliminate heterophilic interference, 10 µL of Heterophilic Blocking Reagent 6 (HBR6, Scantibodies Laboratory) was added to the saliva sample. Finally, 1 µL of both MPAb (1.87×10^9 MP per sample) and Bio-Ab (580 ng per sample) were added and allowed to incubate for at least 5 min but a better signal was achieved for incubations of at least 20 min, which we found to be important for very dilute analyte-spiked samples (recombinant PSSP17 assays). Reactions were added to the feeding pad of gRAD strips, then washed with 120 µL SDB50 for 10 min to allow flow up the strip. Strips were allowed to air dry and develop for at least 5 min. Strips were allowed to dry/develop further dependent on each sample as some samples developed more quickly than others. Test strips were considered negative if the strip had been allowed to dry and develop for more than 30 min with only the control line providing a signal. Considering that the gRAD system will not be the envisioned final diagnostic test design, we estimated the average time from sample acquisition to read out to be between 3-30 mins, including all the incubation times prior to addition to the strip and strip drying. LFIA strips were activated by a LOFTEK® 51 UV LED handheld Flashlight 395 nM and imaged using an amber filter and an iPhone 5S (8 megapixel) camera. Note that Nokia Lumia and Samsung smart phones are also compatible with the LFIA image capture but for the purpose of this study we used the digital camera phone with the lowest resolution.

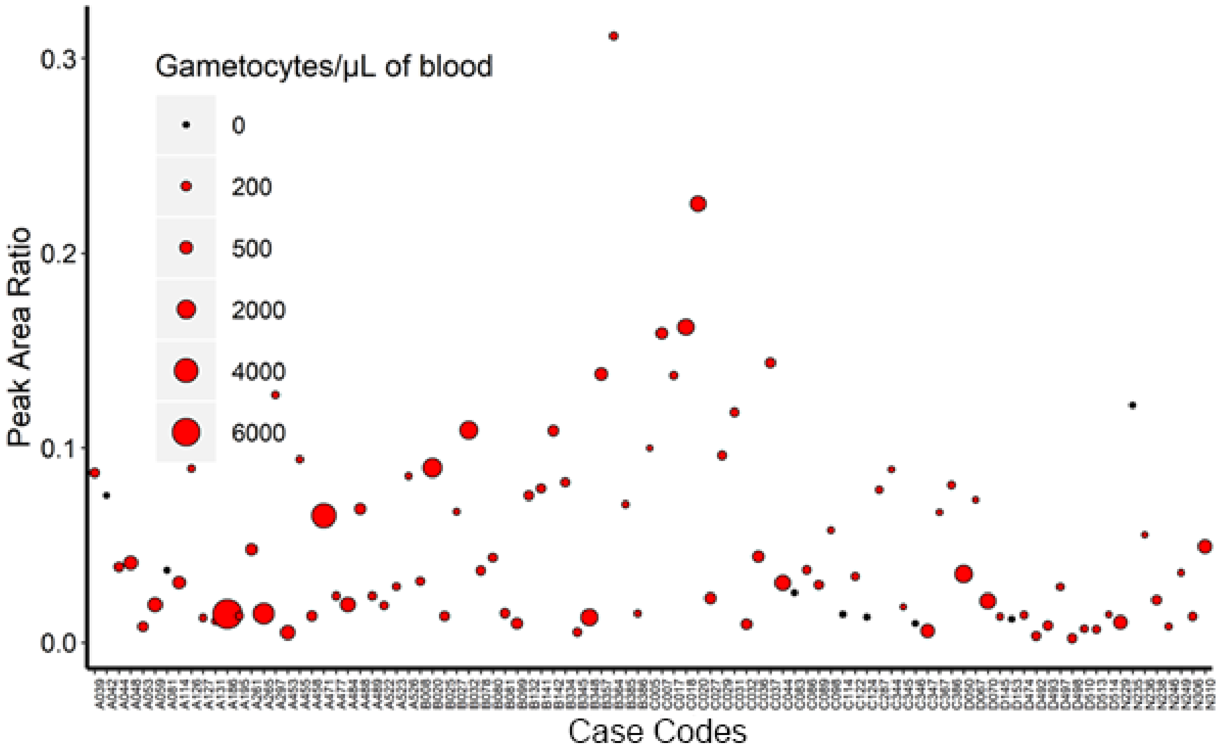

Fig. S1. The correlation of PAR and female gametocyte abundance per microliter of blood (based on *pfs25* transcript number). Point size corresponds to gametocytes per microliter. Case codes are indicated on the x-axis. Only positive Peak Area Ratio (PAR) values are shown in the y-axis, using the PAR = 0.01 as cut-off for positivity of detection of PSSP17.

Table S1. The complete list of *P. falciparum* proteins identified in the saliva from children with subclinical infection in Yaoundé, Cameroon. A total of 35 proteins were identified by LC-MS/MS with a Mascot ions score ≥ 25. Mass Spectrometry evidence for mature gametocytes (G), immature gametocytes (IG) and asexual stages (A), based on the following published references (*20, 23*).

Accession Number	Description	M_r (kDa)	Annotated GO function	MS Evidence
**PF3D7_0310500**	DEAD box helicase, putative	267.38	ATP binding, helicase activity, nucleic acid binding	G
**PF3D7_0401900**	Acyl-CoA synthetase (ACS6)	110.80	long-chain fatty acid-CoA ligase activity	A
**PF3D7_0419900**	Phosphatidylinositol 4-kinase, putative	611.67	phosphotransferase activity	G
**PF3D7_0422300**	Alpha tubulin 2	49.69	GTPase activity, structural molecule activity	A
**PF3D7_0507800**	Conserved *Plasmodium* protein, unknown function	177.43	None	A,G
**PF3D7_0508100**	SET domain protein, putative (SET9)	195.63	zinc ion binding	G
**PF3D7_0509400**	RNA polymerase I (RNAPI)	340.68	DNA binding, DNA-directed RNA polymerase activity	G
**PF3D7_0528700**	Peptidyl-prolyl cis-trans isomerase (CYP23)	23.20	peptidyl-prolyl cis-trans isomerase activity	G
**PF3D7_0610400**	Histone H3 (H3)	15.45	DNA binding	A
**PF3D7_0632500**	Erythrocyte membrane protein 1, PfEMP1 (VAR)	456.92	cell adhesion molecule binding, receptor activity	G
**PF3D7_0704100**	Conserved *Plasmodium* membrane protein, unknown function	425.27	GTPase activity, translation initiation factor activity	G
**PF3D7_0705500**	Inositol-phosphate phosphatase, putative	330.70	None	G
**PF3D7_0717900**	Thioredoxin-like protein	53.38	None	A
**PF3D7_0904100**	Adapter-related protein, putative	161.41	protein binding	G
**PF3D7_0906100**	Developmental protein, putative	21.85	None	A
**PF3D7_0927200**	Zinc finger protein, putative	189.70	RNA binding, zinc ion binding	IG
**PF3D7_1029000**	Conserved *Plasmodium* protein, unknown function, pseudogene	80.89	None	G
**PF3D7_1102400**	Flavoprotein, putative	78.54	None	A
**PF3D7_1105600**	Translocon component PTEX88 (PTEX88)	90.79	None	A
**PF3D7_1134700**	DNA-directed RNA polymerase 1, subunit 2, putative	175.53	DNA binding, DNA-directed RNA polymerase activity, ribonucleoside binding	G
**PF3D7_1207000**	Conserved *Plasmodium* protein, unknown function	311.43	ATP binding, actin binding, calmodulin binding, motor activity	G
**PF3D7_1215100**	Conserved *Plasmodium* protein, unknown function	113.03	ATP binding, actin binding, calmodulin binding, motor activity	IG
**PF3D7_1216000**	Serine--tRNA ligase, putative	73.27	ATP binding, serine-tRNA ligase activity	G
**PF3D7_1216900**	DNA-binding chaperone, putative	111.07	DNA binding, heat shock protein binding	A
**PF3D7_1218800**	Conserved *Plasmodium* secreted ookinete/sexual stage protein, putative	39.63	None	G
**PF3D7_1235700**	ATP synthase subunit beta, mitochondrial	58.40	ATP synthase activity, rotational mechanism, ATPase activity, phosphorylative mechanism	A
**PF3D7_1239900**	vesicle fusion and protein sorting subunit 16, putative (VPS16)	120.30	None	A
**PF3D7_1313500**	Conserved *Plasmodium* membrane protein, unknown function	209.08	hydrolase activity, triglyceride lipase activity	G
**PF3D7_1319200**	Conserved *Plasmodium* protein, unknown function	103.85	flavin adenine dinucleotide binding	G
**PF3D7_1325900**	Conserved *Plasmodium* protein, unknown function	326.15	ATP binding, actin binding, calmodulin binding, motor activity	G
**PF3D7_1327300**	Conserved *Plasmodium* protein, unknown function	205.48	ATP binding, actin binding, calmodulin binding, motor activity	IG
**PF3D7_1337500**	Conserved *Plasmodium* protein, unknown function	393.26	calcium ion binding, receptor activity	IG
**PF3D7_1353000**	Tryptophan-rich antigen, pseudogene	96.25	None	A
**PF3D7_1411400**	Plastid replication-repair enzyme (PREX)	235.82	3'-5' exonuclease activity, ATP binding, DNA binding, DNA helicase activity, DNA-directed DNA polymerase activity	G
**PF3D7_1452200**	Aminomethyltransferase, putative	62.35	None	G

Table S2. LM analyses of blood samples from children (5 to 12 years old) with subclinical infections in Yaoundé, Cameroon. Children were found to be gametocyte- or trophozoite-positive by light microscopy. Pooled saliva from these children was used as sample material for the initial discovery of *P. falciparum*-derived protein markers.

No.	Case Code	Gametocytemia[β]	Parasitemia[β]	Age	Gametocytes/µL	Trophozoites/µL
1	A359	0 gct/1120 WBC	33 asx/500 WBC	10	0	528
2	C159	118 gct/µL	5 asx/505 WBC	6	944	79
3	C160	210 gct/µL	11 asx/501 WBC	9	1,680	176
4	C161	5 gct/µL	1 asx/500 WBC	8	40	16
5	C162	24 gct/µL	29 asx/500 WBC	11	192	464
6	C163	4 gct/µL	367 asx/524 WBC	11	32	5,872
7	C164	380 gct/µL	58 asx/506 WBC	11	3,040	917
8	C165	108 gct/µL	0 asx/500 WBC	8	864	0
9	C166	6 gct/µL	162 asx/511 WBC	7	48	2,536
10	C167	36 gct/µL	32/500 WBC	10	288	512
11	C168	65 gct/µL	83/504 WBC	8	520	1,317
12	D162	0 gct/1560 WBC	403 asx/208 WBC	10	0	15,500

[β]Gametocyte (gct); Asexual (asx); White Blood Cell (WBC). For gametocytes, the count is against 1,000 WBC unless otherwise noted below. For trophozoites (asx), the count is against 500 WBC unless otherwise noted.

Table S3. Description of samples collected from schools in Cameroon.

Table S4. Description of samples collected from homes in Zambia.

Table S5. Quantification of gametocytes per µl of blood in a subset (*n* = 100) of samples from Cameroon.
